# Texas’ Sin of Omission

**Published:** 1899-01

**Authors:** 


### Texas' Sin of Omission.—Her Sanitary Needs.

A distinguished sanitarian has said, "every death from a preventable disease is a crime."

It *is* a crime, chargeable to the *State,* and not to the doctors, for this State, at least, has refused to hear the voice of the medical profession, which for twenty years has been urging upon the Legislature the necessity of sanitation, in a broad sense, and sounding in their ears the warning that prevention is not only better, but far cheaper than *cure.* Time and again have the great principles of State medicine and public hygiene been preached to them by the medical profession, but it seems we had as well sing psalms to a deaf man—for all the impression it has ever made or results it has ever brought. The average legislator has seemed to have no conception of "economy" broader than the reduction of government expenses—by cutting down the appropriation for even the working machinery of the government. The medical profession—represented in the person of committees of experienced physicians appointed by our State Medical Association, have in vain sought to impress the truth upon legislators that a little money expended in public sanitation,—in investigating and *removing the cause of diseases* which annually destroy thousands of lives, would, like bread cast upon the waters, be returned after many days, tenfold increased; would result in a saving of life, a saving of time from sickness, which would add a thousand fold to the material prosperity and wealth of the State. The people need to be educated up to the point of seeing the matter in that light, and the legislators made to see that true economy lies in the protection of the public health—not alone from *imported* diseases.

In the application of the principles of State medicine, the State occupies the same relation to the public that the individual practitioner does to his clientelle; the State should apply the general principles of sanitation in every community, as the practitioner does in each family of his patrons. No intelligent physician, when called to a case of typhoid fever, diphtheria or other preventable disease, will be content to deal with the *result of causes;*—to treat disease which has been caused by insanitary environment, lack of drainage, want of ventilation, filth, seepage water, sewer exhalations or what not,—but will seek the cause and *have it removed.* (The true principle which should be inculcated and practiced would be to seek and remove the cause before the first case occurs.) And this is the principle which the medical profession has sought for years, and is now seeking, to impress upon those who, alone, have the authority to institute the necessary measures of prevention, and provide the necessary means to enforce them.

It is a reproach to the boasted enlightenment of a great State like Texas that sanitation is so totally neglected. We are the laughing stock of all other States. The collection and preservation of our vital statistics,—the fundamental factor in all sanitary reform,—for no progress can be made in prevention till the number of deaths and the causes of the same are known,—is totally neglected. It is humiliating in the highest degree to be compelled, when interrogated, as the writer often is, by sanitarians, United States government officials, editors of other journals, etc., to answer that in Texas there is no Public Health Department, no Board of Health, no record of vital and mortuary statistics, no attention whatever paid by the State to sanitation in any of its branches; no statistics of water supply, no measures to prevent the pollution of streams nor the adulteration of foods; no protection afforded the people against the sale of the meat of diseased animals, etc. There are no records kept from which any person can get any idea of how many persons die in Texas every year; nor how many die of typhoid fever, consumption, diphtheria and the numerous other diseases that ought to be prevented. Such is the case in glorious Texas.

True, we have a quarantine department, and the sanitary administration of the State government is confined solely to that function—the prevention of the introduction into the State, from without, of diseases which should, if the general government did its duty intelligently and efficiently, have never been permitted to be imported to our shores; diseases which, if enlightened sanitation were enforced in their habitat, would *never have occurred.* Every effort is yearly directed to this one thing, and the sum total of the meagre appropriation for the quarantine department has been expended in this one function, a sum barely sufficient to meet the known expenses of keeping up six coast and three border stations. For the want of sufficient means to employ such methods as would enable this department to conduct even a modified quarantine;—to facilitate commerce, at least by permitting such merchandise to be brought into the State as is definitely known to be incapable of carrying yellow fever infection,—this officer has been compelled to enforce absolute non-intercourse with the outside world, thus paralyzing commerce, and exposing the State to such jeers as the "senseless Texas quarantine," and to the reproach of a New Orleans paper, recently, that "Texas put the embargo on a flat car of pig iron." (Fact.) The State has neglected most shamefully to make any provision for the prevention of those deadly diseases, the result of local causes, which annually destroy more lives than all the yellow fever epidemics combined.

In brief, Texas *needs something more than a quarantine department!* That the Texas quarantine has, in the past, been as efficiently administered as the circumstances and the means at the disposal of its head would admit, is hardly open to question; however questionable its methods may be thought to have been,—that is, if we are to judge solely by results, obtained at whatever cost,—the fact that while Louisiana and Mississippi have suffered visitations of (alleged) yellow fever, Texas escaped. But, was the game worth the candle, considering the small mortality of the last two epidemics (an average of less than four per cent., we believe), the cost of keeping it out of Texas, to the neglect of preventive measures against deadly local diseases?

Because the writer was, for several years, connected with the quarantine department of Texas; because he opposed its transfer to the United States Marine Hospital Service, and was loyal to the State administration, let it not be supposed that he holds a brief for its defense, or that he is blind to its shortcomings. By reason of that connection he knows, better than others, perhaps, its defects and its needs. They have often been pointed out to the Legislature and to governors, but neither governor nor law-maker has heretofore seemed to be much impressed, either with the necessity of preserving our vital statistics, or instituting measures of prevention to lessen the death rate of indigenous diseases,—diseases generated by filth, for the most part. Time and again have they been petitioned by the medical profession to create a rational health department, and in nearly every report the late distinguished State Health Officer has asked for the services of at least a chemist and bacteriologist. Their purblind vision has apparently not risen to a conception of sanitation beyond the function of quarantine.

Undismayed by repeated refusals, the State Medical Association will, this session, make one more effort to secure the passage of a Board of Health bill. The incoming governor is an enlightened statesman,—an old congressman,—and hopes are entertained of impressing him with the necessity of more security to the public health than is afforded by a bomb-proof quarantine against the introduction of foreign infection, at a cost of annually closing our ports to the world six months of every year. It is understood that he has invited a free discussion of the subject of public sanitation, and the necessity of a change in the existing system; that he wishes to get the opinion of the medical profession on the subject. This explains why he has not, to date, indicated his choice for State Health Officer. It would seem, too, to warrant the hope that he will heed the wishes of the medical profession, so often urged in vain, and will make his recommendation to the Legislature accordingly as to public health matters.

We believe the JOURNAL voices the unanimous opinion of the medical profession when we say, *Texas needs a Public Health Department,* for the administration of all the functions of State sanitation. Such department or Board of Health should have a quarantine officer, a bureau of vital statistics in charge of a superintendent, a pathologist (at once microscopist, chemist and bacteriologist), and at least two yellow fever experts as sanitary inspectors. In each county there should be a bonded medical officer who should have sanitary supervision of the county and to whom all physicians and midwives should, under penalty for failure, report all births and deaths, and the causes of death, etc.

But this is a mere detail. The one thing to be done *now* is to show Mr. Sayers the wisdom and importance of asking the Legislature to create a department or Board of Public Health in lieu of the present very limited and primitive institution.

We suggest that the Texas physicians, on reading this, at once write to Governor Sayers, urging his attention to the crying needs of enlightened sanitary legislation.

In pursuance of the foregoing, for the information of our readers, and of the Governor and Legislature, and as showing the value of sanitation as a life-saving institution, we reproduce the following from Professor Andrew D. White's great work, *"The Warfare of Science with Theology,"* article entitled:

"THE TRIUMPH OF SANITATION."

"The recent history of sanitation in all civilized countries shows triumphs which might well fill us with wonder, did there not rise within us far greater wonder that they were so long delayed. Amazing is it to see how near the world has come again and again to discovering the key to the cause and cure of pestilence. It is now a matter of the simplest elementary knowledge that some of the worst epidemics are conveyed in water. But this fact seems to have been discovered many times in human history. In the Peloponnesian war the Athenians asserted that their enemies had poisoned their cisterns; in the Middle Ages the people generally declared that the Jews had poisoned their wells; and as late as the cholera of 1832 the Parisian mob insisted that the water-carriers who distributed water for drinking purposes from the Seine, polluted as it was by sewage,* had poisoned it, and in some cases mur-

---

[*At Austin, the Capital of Texas, the home of the Governor and of the State Health Officer, and of an able medical profession (who were not consulted), last spring, during the rainy season—five regiments, (5000 men and 1000 horses), were camped more than 30 days on a site near the city, which necessarily drains into the artificial lake formed by a granite dam costing Austin, in round numbers, some $2,000,000—the storage-tank of Austin's water supply. That was before the filters were put in by the city, and this water—muddy from the freshets of spring, and, of course, reeking from the germs of disease from the scourings of a military camp—was pumped into our houses and the camp for domestic purposes. The writer ventured to call attention to the matter in a note to the *Austin Daily Statesman*, requesting it to urge all families using this water to have it boiled and filtered, but this note was judiciously suppressed for fear of injuring the city. That Austin escaped a scourge is as remarkable as it is fortunate.—ED.]

dered them on this charge. Had not such men as Roger Bacon and his long line of successors been thwarted by theological authority,—had not such men as Thomas Aquinas, and Vincent of Beauvais, been drawn or driven from paths of science into the dark, tortuous paths of theology, leading no whither,—the world to-day, at the end of the nineteenth century, would have arrived at the solution of great problems and the enjoyment of great results, which will only be reached at the end of the twentieth century, and even in generations more remote. Diseases like typhoid fever, diphtheria, pulmonary consumption, scarlet fever, pneumonia and *la grippe,* which now carry off so many most precious lives, would have long since ceased to scourge the world.

"The recent history of hygiene in all countries shows a long series of victories, and these may well be studied in Great Britain and the United States. * * * It was only in the year 1838 that a systematic sanitary effort was begun in England by the public authorities. The state of things at that time, though by comparison with the Middle Ages, happy, was, compared with what has since been gained, fearful; the death rate among all classes was high, but among the poor it was ghastly. Out of seventy-seven thousand paupers in London during the years 1837-8, fourteen thousand were suffering from fever, and of these, nearly six thousand from typhus. In many other parts of the British islands the sanitary conditions were no better. A noble body of men grappled with the problem, and in a few years one of them rose above his fellows—the late Edward Chadwick. The opposition to his work was bitter. * * * Chadwick began to be widely known in 1848 as a member of the Board of Health, and was driven out for a while he fought the opposition, developed the new work, and one of the for over-zeal; but from one point or another, during forty years, he fought the opposition, developed the new work, and one of the best exhibits of its results is shown in his address before the Sanitary Conference at Brighton in 1888. From this and other perfectly trustworthy sources some idea may be gained of the triumph of the scientific over the theological method of dealing with disease, whether epidemic or sporadic.

"In the latter half of the seventeenth century the annual mortality of London is estimated at not less than eighty in a thousand; about the beginning of this (19th) century, it stood at twenty-four in a thousand; in 1889 it stood at less than eighteen in a thousand; and in many parts, the most recent statistics show that it has been brought down to fourteen or fifteen in a thousand. * * *

"The Public Health Act having been passed in 1875, the death rate in England, among men, fell, between 1871 and 1880, more than four in a thousand, and among women more than six in a thousand. In the decade between 1851 and 1860 there died of diseases attributable to defective drainage and impure water, over four thousand persons in every million throughout England; these numbers having declined till in 1888, there died less than two thousand in every million. The most striking diminution of the deaths from such causes was found in 1891 in the case of typhoid fever, that diminution being fifty per cent. * * * As to the scourge which, next to the Black Death, was formerly the most dreaded—small pox—there died of it in London during the year 1890, just one person.

"Drainage in Bristol reduced the death rate by consumption from 4.4 to 2.3; at Cardiff, from 3.47 to 2.31; and in all England and Wales, from 2.68 in 1851, to 1.55 in 1888.

"What can be accomplished by better sanitation is also seen to-day by a comparison between the death rate among the children outside and inside the charity schools. The death rate among those outside was, in 1881, twelve in a thousand; while inside, where the children were under sanitary regulations, maintained by competent authorities, it has been brought down, first, to eight, then to four, and finally to less than three in a thousand.

"Statistics carefully kept show [in France—Ed.], that the mean length of human life has been remarkably increased. In the eighteenth century it was but twenty-three years; from 1825 to 1830 it was thirty-two years and eight months; and since 1864, thirty-seven years and six months.

"Nor has the recent history of the United States been less fruitful in lessons. Yellow fever, which formerly swept not only Southern cities but even New York and Philadelphia, has now been almost entirely warded off. Such epidemics as that in Memphis in 1878, and the immunity of that city from its visitations since its sanitary conditions were changed by Mr. Waring [the abandonment of the cess-pool system and the substitution of the Waring system of sewers, and the Holly system of water-works—Ed.] are a most striking object lesson to the whole country.* * * * Cholera, which again and again swept the country, has ceased to be feared by the public at large."

---

THAT PART OF THE QUARANTINE ACT now in force in Texas which gives each county the authority to quarantine against any and all other counties or places "whenever the county commissioners have reason to fear invasion of infection from such county or place," independently of the State Health Officer, ought to be repealed. This authority is a license to enforce shot-gun quarantine and stop trains by local authorities without reference to the views or authority of the State Health Officer. True, he can cause such quarantine to be raised if he does not approve of it, but it takes time, gives trouble and occasions loss and inconvenience, meantime.

THAT CLAUSE referred to elsewhere, which obliges the State to feed and lodge persons detained at quarantine camps on our border ought also to be repealed. Persons who arrive at such stations and find they cannot enter the State should go back, or go to some hotel near by,—not, of course, on the Texas side,—and pay their own expenses. In most cases such persons know before they start on the trip that they will run up against a Texas quarantine; it is incident to travel; so is a wreck, or a missed connection or a detention from any other cause. In the event of detention from any cause *except quarantine,* no traveler expects another party to pay his expenses. It is absurd that the tax payers of the State should have this expense to meet. It is as good a thing as a tramp or a score of them would want. The State paid one bill of $264 for board and lodging of a party of refugee Cubans stopped at one of the Texas border stations on the Rio Grande, and a well-to-do citizen of San Antonio, returning home through Mexico, was similarly entertained at State expense. The quarantine act should be overhauled and repaired.

---

ABSOLUTE QUARANTINE, non-intercourse with the rest of the world, the tieing up of the wheels of commerce, is a relic of barbarism; it is out of date, and should no longer find place in the economics of an enlightened people. The very name is obsolete.

*According to the report of the city physician of Memphis, Dr. G. B. Thornton, published in 1880, this improved sanitation in one year reduced the mortality from all zymotic diseases—local—fifty per cent.

It is of French origin, and is derived from *"quarante"*—forty, and means to tie up or prohibit infection for forty days; hence, to "quarante," or "quarantine" any person or thing for less than forty days, is a misapplication of the term. In light of the revelations of bacteriology, and the germ causation of epidemic diseases, of antisepsis, and in knowledge of the almost exact period of incubation of most diseases, it would now be considered barbarous to hold a vessel forty days—especially, as was the case in old times—unless, meantime, she were "disinfected" and cleansed.

The entire method should be reversed. Instead of shutting all ports against the introduction of infection, the infection should be isolated, surrounded and exterminated. Should a fire break out in a city what would be thought of the policy of leaving it to spread everywhere, while all the people would go home and shut their doors? Yellow fever is an exotic; it never originates in America, and when introduced here (which should never be permitted), it can only spread in unsanitary environments. A system of international inspection of all trade and travel should be instituted, and persons and things from yellow fever, cholera or plague centers should not be permitted to embark or sail for America. Those who have read Dickens' "Uncommercial Traveler,"—the article, "An Emigrant Ship,"—will understand what I mean. A ship with eight hundred emigrants for America was anchored in the Mersey ready to sail. The doctor, the sanitary inspector of England (that was forty years ago, too), went on board and personally inspected every man, woman and child and their belongings. Satisfying himself that there was no infection on board, and that the sanitary condition of the boat and cargo was such as to not generate disease, he then vaccinated all who were not protected, and let her go.

Were measures of the kind instituted now and honestly and faithfully carried out, as is still the case with Great Britain, there would be no necessity for the Southern States to close their ports six months out of every year. We surely are a primitive people in Texas.

---

To ADMINISTER QUARANTINE, solely, Texas does not need a Board of Health. I maintain that that function can best be discharged by one man, provided he is a capable man, and that means a good deal. For when action is to be taken it should be prompt, and circumstances arise during the prevalence of epidemics which will not admit of delay even for consultation.

But, as said elsewhere, quarantine is not all of sanitation, and

Texas needs a Board of Health, or a Health Department, or a Commission, call it what you will, that shall be empowered and enabled, by a suitable appropriation, to institute and execute all the various measures for the prevention of disease and death, the better protection of the public health and the commerce of the State.

This subject is not understood by the people, nor by governors and legislators;—they have not given it thought.

For an understanding of the requirements, at this opportune time, when the medical profession will submit the needs of sanitary reforms to the Legislature, we may be pardoned for enumerating here, what is merely elementary knowledge with sanitarians, some of the principal functions pertaining to sanitation or preventive medicine:

Quarantine.—And it should be *rational,* and not absolute non-intercourse; and ample means for the purpose should be available.

Compilation and preservation of vital and mortuary statistics. Every birth should be recorded, and every death and the cause of it, should also be recorded.

The unsanitary conditions of life and environment, so fruitful of disease and death, should be studied, and these can never be known and removed until we compile the vital statistics of the people.

Sanitary supervision of cities and towns, and of the dwellings and places of business of the people, factories and shops, schools, slaughter houses, etc.; of the slaughter of animals for food, and of the carcasses of such sold for food, as is the case even in Mexico. There every animal is passed upon by a government officer before it is killed, and the meat is stamped, numbered and a record kept of it.

The institution of drainage, the abolition of the cess-pool system in all communities; the protection of all streams and other sources of drinking water from pollution; the prevention of adulteration of food, etc.; the instruction of the people by publication, in the hygiene of living; the introduction of the general and special principles of sanitation in schools, prisons and factories; of compulsory vaccination, etc.; in short, the inculcation of such of the principles of sanitary science and the universal institution of such measures as will tend to lessen the death rate, and consequently add to the average length of life, and augment the material wealth and prosperity of the State.

---

THE AUSTIN DISTRICT MEDICAL SOCIETY held its 34th quarterly meeting at Austin December 22 (ult.). The following papers were read and discussed:

"Chronic Cystitis in the Aged."—I. J. Jones, M. D., Surgeon Confederate Home, Austin.

"Stone in the Urinary Bladder—Report of a Case."—A. N. Denton, M. D., Austin.

"Then and Now in Medical Practice."—D. M. Reagan, M. D., Kyle.

"Placenta Prævia—Report of a Case."—M. M. Smith, M. D., Austin.

"Diagnosis and Treatment of Gall Stones."—W. J. Mathews, M. D., Austin.

Officers elected:

President, Dr. J. C. Anderson, Granger, Texas.

First Vice-President, Dr. H. B. Hill, Austin, Texas.

Second Vice-President, Dr. J. S. Watson, Manor, Texas.

Secretary and Treasurer, Dr. S. E. Hudson (holds over), Austin, Texas.

The doctors were entertained by the resident profession at an elegant dinner at 8 o'clock at the Driskill, where all went as merry —(not as a marriage bell, excuse me)—as a lot of good natured, jolly and agreeable doctors can make such occassions—(and there was *no* "flowing bowl").

